# A Review of the Mycotoxin Enniatin B

**DOI:** 10.3389/fpubh.2017.00304

**Published:** 2017-11-16

**Authors:** Alessandra Prosperini, Houda Berrada, María José Ruiz, Francesca Caloni, Teresa Coccini, Leon J. Spicer, Maria Chiara Perego, Alessandra Lafranconi

**Affiliations:** ^1^Laboratory of Toxicology, Faculty of Pharmacy, University of Valencia, Valencia, Spain; ^2^Department of Veterinary Medicine (DIMEVET), Università degli Studi di Milano, Milan, Italy; ^3^Laboratory of Clinical and Experimental Toxicology, Toxicology Unit, Maugeri Clinical Scientific Institutes SpA-BS, IRCCS Pavia, Pavia, Italy; ^4^Department of Animal Science, Oklahoma State University, Stillwater, OK, United States; ^5^Centro di Studio e Ricerca sulla Sanità Pubblica (CESP), Università Milano Bicocca, Milan, Italy; ^6^Department of International Health, FHML, CAPHRI, Maastricht University, Maastricht, Netherlands

**Keywords:** enniatin B, toxic effects, biological properties, biochemical activities, emerging findings

## Abstract

Mycotoxin enniatin B (ENN B) is a secondary metabolism product by *Fusarium* fungi. It is a well-known antibacterial, antihelmintic, antifungal, herbicidal, and insecticidal compound. It has been found as a contaminant in several food commodities, particularly in cereal grains, co-occurring also with other mycotoxins. The primary mechanism of action of ENN B is mainly due to its ionophoric characteristics, but the exact mechanism is still unclear. In the last two decades, it has been a topic of great interest since its potent mammalian cytotoxic activity was demonstrated in several mammalian cell lines. Moreover, the co-exposure *in vitro* with other mycotoxins enhances its toxic potential through synergic effects, depending on the concentrations tested. Despite its clear cytotoxic effect, European Food Safety Authority stated that acute exposure to ENNs, such as ENN B, does not indicate concern for human health, but a concern might be the chronic exposure. However, given the lack of relevant toxicity data, no firm conclusion could be drawn and a risk assessment was not possible. In fact, very few studies have been carried out *in vivo* and, in these studies, no adverse effects were observed. So, research on toxicological effects induced by ENN B is still on-going. Recently, some studies are dealing with new advances regarding ENN B. This review summarizes the information on biochemical and biological activity of ENN B, focusing on toxicological aspects and on the latest advances in research on ENN B.

## Introduction

*Fusarium* species^1^ are common pathogens of cereal grains, animal feeds, and food commodities worldwide (1). Under favorable conditions, their secondary metabolism can produce hexadepsipeptidic mycotoxins,^2^ such as enniatins (ENNs). ENNs are commonly found in several grains and their derived products, in fish, dried fruits, nuts, spices, cocoa, coffee products, etc. (2–7). Moreover, some food processes including cooking, baking, frying, roasting, etc. do not affect their chemical structure; so, detoxification strategies to mitigate the risks of ENNs presence in foods and feed may be difficult (8, 9).

**Figure 1:**
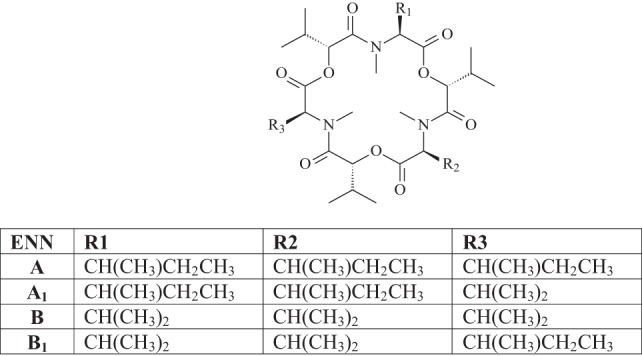
Chemical structure of enniatins (ENNs).

Structurally, ENNs are cyclohexadepsipeptides composed of alternating residues of three N-methyl amino acids, commonly valine, leucine, and isoleucine, and three hydroxy acids, typically hydroxyisovaleric acid. Several ENNs analogs (A, A1, B, B1, B2, B3, B4, D, E, F, and G) have been identified. Among them, the most prevalent ENNs reported as natural contaminants in cereals in Europe are ENN A, A1, B, and B1 (10). Their chemical structure is reported in Figure 1.

The lipophilic nature of ENNs allows them to be incorporated into lipid bilayers of cell membranes and creates cation selective pores that cause an increase in the permeability for cations, resulting in disturbances of the physiological cation level in the cell (11). Their ionophoric^3^ behavior seems to be related to their wide range of biological activity. ENNs are known to be insecticidal, antifungal, antibacterial, and antihelmintic (12). Moreover, they exerted a potent cytotoxic effect in several human and animal cell lines at very low micromolar range (10, 13–18). Despite the strong cytotoxicity *in vitro*, a few studies carried out *in vivo* did not show relevant toxicity (19–23).

Unlike other *Fusarium* mycotoxins, such as deoxynivalenol (DON), T-2, HT-2, fumonisins (FB), and zearalenone (ZEA), whose presence in food and feed has been regulated by authorities, no limits have been set for ENNs, up to now. However, an increasing number of studies are proving their presence in several food and feed commodities and also their toxicity (2). This fact may constitute a great concern for human and animal health, since their toxicity could be also enhanced by the presence of other mycotoxins at the same time. The European Commission asked the European Food Safety Authority (EFSA) for a scientific opinion on the risks to human and animal health related to the presence of ENNs in food and feed. EFSA concluded that acute exposure to ENNs does not indicate concern for human health. There might be a concern with respect to chronic exposure, but no firm conclusion could be drawn and a risk assessment was not possible for dietary exposure to ENNs, due to the overall lack of toxicity data (24). At the moment, EFSA is still collecting occurrence data for a future risk assessment.

Among the four ENNs above-mentioned, ENN B is currently the most studied since it has been the most-often detected in unprocessed and processed grains from European countries. Concentrations of ENN B in grains range from a few μg/kg to over mg/kg (12). In a multi-mycotoxins analysis of maize silage in NW Spain, Dagnac et al. (25) found that ENN B was the most prevalent mycotoxin detected in 51% of the samples (average concentration: 157 µg/kg). Similar ENN B concentrations (195.5 ± 47.0 µg/kg) were observed in cereal samples collected from European and African countries (26). Svingen et al. (27) demonstrated the ENN B presence in all of the samples of Danish grain collected during the 2010 and 2011 harvests, with the highest value of 3,900 µg/kg detected in rye sample. A survey in Finland showed that ENNs were frequently detected in unprocessed grains including wheat, barley, rye, and oats, and that the maximum concentration was found for ENN B (10,280 µg/kg) in a barley sample (28).

Regarding grain-based products, in pasta samples bought from Dutch shops, de Nijs et al. (8) found the highest incidence for ENN B with concentrations ranging from 7.0 to 175 µg/kg. Higher concentrations of ENN B (up to 1,100 µg/kg) was detected in pasta and baby food from Italian supermarkets by Juan et al. (2). Zinedine et al. (7) demonstrated that wheat couscous semolina has a higher ENN B incidence and concentration (592 ng/g) than barley (50 ng/g) or corn (57 ng/g) semolina couscous. In beer samples from Germany, ENN B was the only ENN detected (0.9 µg/L) showing increased incidence than other mycotoxins (29).

Therefore, the attention on ENN B toxicological aspect is still highly concerning, considering that its potential toxicity may be enhanced by co-occurrence with other ENNs or other mycotoxins (15, 30, 31).

Besides its ionophoric property, ENN B toxicity involves the inhibition of acyl-CoA: cholesterol acyl transferase (ACAT) activity^4^ (32) and oxidative stress^5^ (16). ENN B also exerts cytotoxic activities by inducing mitochondrial modifications and cell cycle disruption, finally resulting in apoptotic cell death (16, 33–35). Moreover, it produces adrenal endocrine toxicity (36). A recent study reports a potential anticancer activity (37).

The objective of this review is to compile the effects produced by the *Fusarium* mycotoxin ENN B, focusing on its biological properties, biochemical activity and *in vitro* toxicological effects including the latest research on ENN B, in terms of biological properties, biochemical activity, and toxicity.

## Biological Properties of ENN B

Enniatin B exhibits a wide array of biological activities. Several studies investigated the insecticidal activity of ENN B individually and in complex with other ENNs (38–42). This activity has been confirmed in the blowfly *Calliphora erythrocephala*, in the mosquito larvae (*Aedes aegypti*), in the spruce budworm (*Choristoneura fumiferana*) and against the plant-parasitic nematode *Meloidogyne javanica* (38–40). Moreover, ENN B partially inhibited spore germination of *B. cinerea* (42). However, no insecticidal activity of ENN B was found by Mulè et al. (43) against larvae of *Galleria mellonella*.

Enniatin B exhibits antibacterial activity against some pathogens of humans, such as *Escherichia coli* (CECT 4782), *Enterococcus faecium* (CECT 410), *Salmonella enterica* (CECT 554), *Shigella dysenteriae* (CECT 584), *Listeria monocytogenes* (CECT935), *Yersinia enterocolitica* (CECT 4054), *Clostridium perfringens* (CECT 4647), *Pseudomonas aeruginosa* (CECT 4628), and two strains of *Staphylococcus aureus* (CECT 240 and CECT 976) (44). Moreover, antibacterial effect of ENN B has been demonstrated against *Mycobacterium phlei* and *M. paratuberculosis* (45–47).

On the other hand, ENN B acts also as antifungal agent for *Beauveria bassiana* (CECT 20499, CECT 20191, CECT 20412) and *Trichoderma harzianum* T22 (48). A mixture of ENNs (ENN A, A1, B, and B1 in ratio 5:15:35:45) caused necrotic lesions in potato tuber tissue (49) and ENN B on knapweed leaves (*Centaurea maculosa*) when exposed with acetamido-butenolide (50). Combination of ENN A + ENN B showed decreased leaf and root development, wilting of shoots, necrosis of leaves, and loss of turgor (51, 52).

## Biochemical Activity of ENN B

### Ionophoric Properties

**Figure 2:**
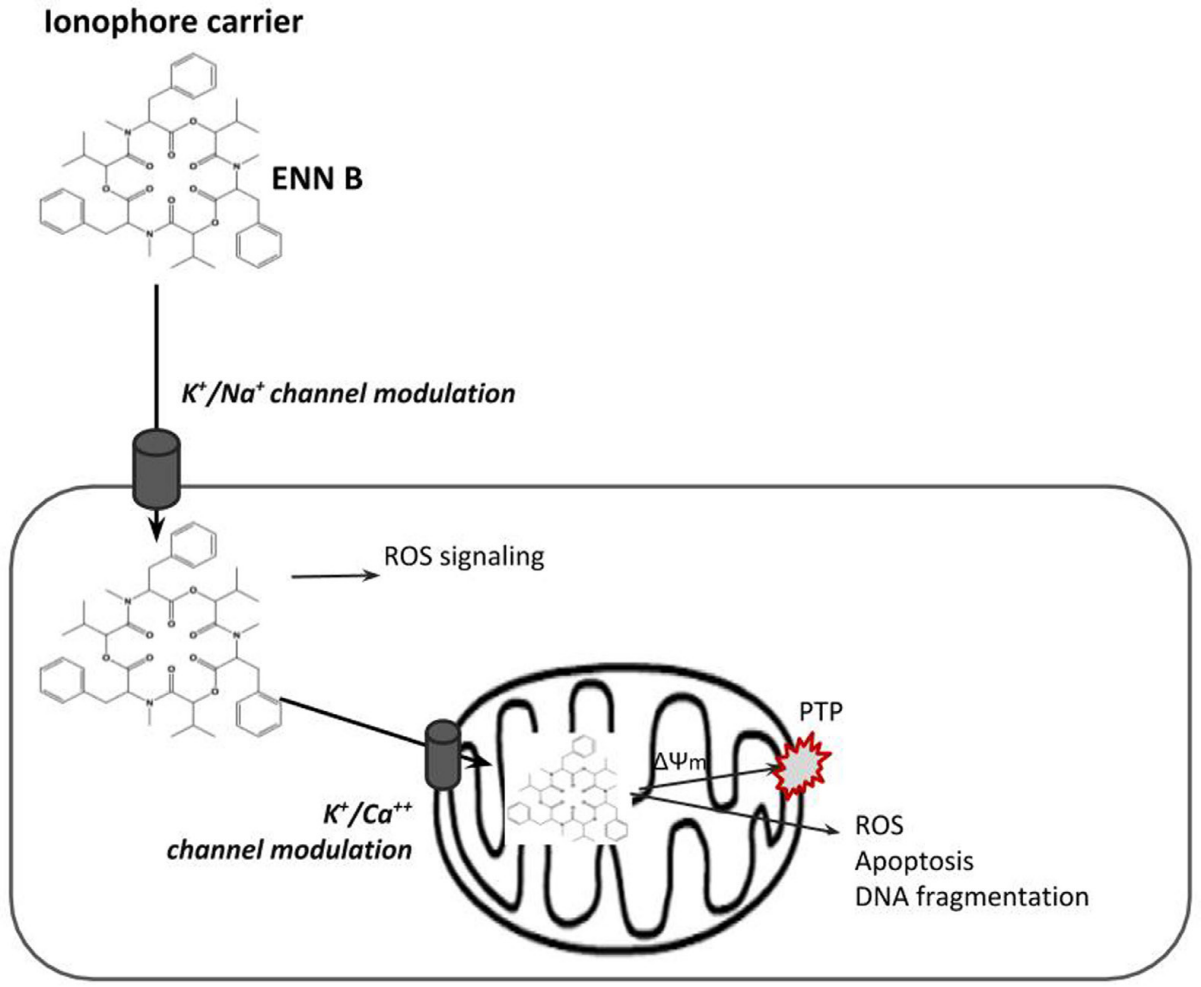
Scheme of the ionophore enniatin (ENN) B carrying ions by diffusing through membrane lipids.

The ionophoric property of ENNs allows them to be capable of promoting the transport of mono- and divalent cations through membranes leading to toxic actions via disturbances in their normal physiological concentrations (1). The primary action is the ionophoric property, which enables ENNs to form stable complexes with cations, and transport them into the lipophilic phase (1) evoking changes in intracellular ion concentration, disrupting cell functions (Figure 2) (53).

The ability of the ENNs to form complexes with alkali metal ions and increase the cationic permeability of membranes has been previously documented (54, 55). In particular, cations transported by ENNs in liposome seems to involve a mobile carrier mechanism which is selective for K^+^ versus Na^+^, requiring two ENN molecules, and it is realized by a “sandwich” model (56). ENNs form both 1:1 and 2:1 ENN:cation complexes with alkali, alkaline earth, and various transition metal ions. The probability of the 3:2 conformations is much less than the two other conformations (57). It has been suggested that electronic, inductive, or steric effects could indirectly stabilize the 2:1 complex. Cation selectivity was ranked as follows: K^+^ > Ca^2+^ ≥ Na^+^ > Mg^2+^ > Li^+^ (56). In addition, the transport efficiency appears to be related to the hydrophobic trait of the ENN molecules. The largest conductivity was shown for ENN B, followed by ENN A1 and B1 (56).

The mitochondriotoxic properties of ENNs have been demonstrated in isolated rat mitochondria (11). The mitochondrial effects were strongly connected with the K^+^ ionophoric activity with ENNs inducing K^+^ uptake by mitochondria. Moreover, they decreased the calcium retention capacity of the mitochondrion matrix leading to the mitochondrial membrane potential (MMP) collapse via permeability transition pore (PTP) opening (11, 58).

### Enzyme Inhibitor

The inhibition of the activity of ACAT by ENN B has been demonstrated (32). Such inhibition could be significant in the treatment and prevention of atherosclerosis and hypercholesterolemia. Trenin et al. (59) showed strong hypolipidemic activity of ENN B in human hepatoma HepG2 cells as a result of the inhibition of ACAT activity, triglyceride biosynthesis, and diminished pool of free fatty acids in the cells.

### Other Biochemical Properties

Enniatin B was the most effective inhibitor of one of the major multidrug efflux pumps such as Pdr5p^6^ in *Saccharomyces cerevisiae* at non-toxic concentrations (60). The inhibition mechanism is clearly different from its function as an ionophore (60). This ENN B property may be important for the clinical use in combination with chemotherapeutic drugs.

Enniatins interact with membrane-located ATP-binding cassette (ABC) transporters^7^, especially with ABCB1 and ABCG2 transporters, suggesting potential influences on bioavailability of xenobiotics and pharmaceuticals (61).

## Toxicity of ENN B

Few toxicological studies of ENN B have been performed *in vivo*. Table 1 illustrates *in vivo* studies carried out with ENN B alone and in combination with other ENNs. *In vivo* toxicokinetic trials using pigs demonstrated a higher bioavailability of 91% for ENN B (62). Interestingly, Rodríguez-Carrasco et al. (22) found no acute toxicity in mice after intraperitoneal administration, although ENN B bioaccumulation in the lipophilic tissues was observed. According to Fraeyman et al. (63), ENN B was readily distributed to broiler chicken tissues, with mean volumes of distribution of 33.91 L/kg.

**Table 1:** *In vivo* toxicity studies of enniatin B (ENN B).

Animal	Dosage/route	Effects	Reference
Broiler chicken	0.2 mg/kg b.w.	Tissue bioaccumulation	Fraeyman et al. (63)
	Bolus		

Mice	5 mg/kg b.w.of ENN B	No acute damage	Rodríguez-Carrasco et al. (22)
	Intraperitoneal on two consecutive days	Tissue bioaccumulation	

Mice	1.25–40 mg/kg b.w. (every 8 h)	Mice died in 2–5 days (10–40 mg/kg b.w.)	McKee et al. (19)
	Intraperitoneal	Reduction of weight	
		No anti-HIV activity	

Pigs	0.05 mg/kg b.w.	Absorption	Devreese et al. (62)
	Oral bolus	ENN B > B1 > A1 > A	

Wistar rats	Mixture of ENNs containing 1.19, 2.16, 1.03 and 1.41 mg/kg b.w.of ENN A, A1, B, and B1	No adverse effect	Escrivá et al. (23)
	Oral		

**Figure 3:**
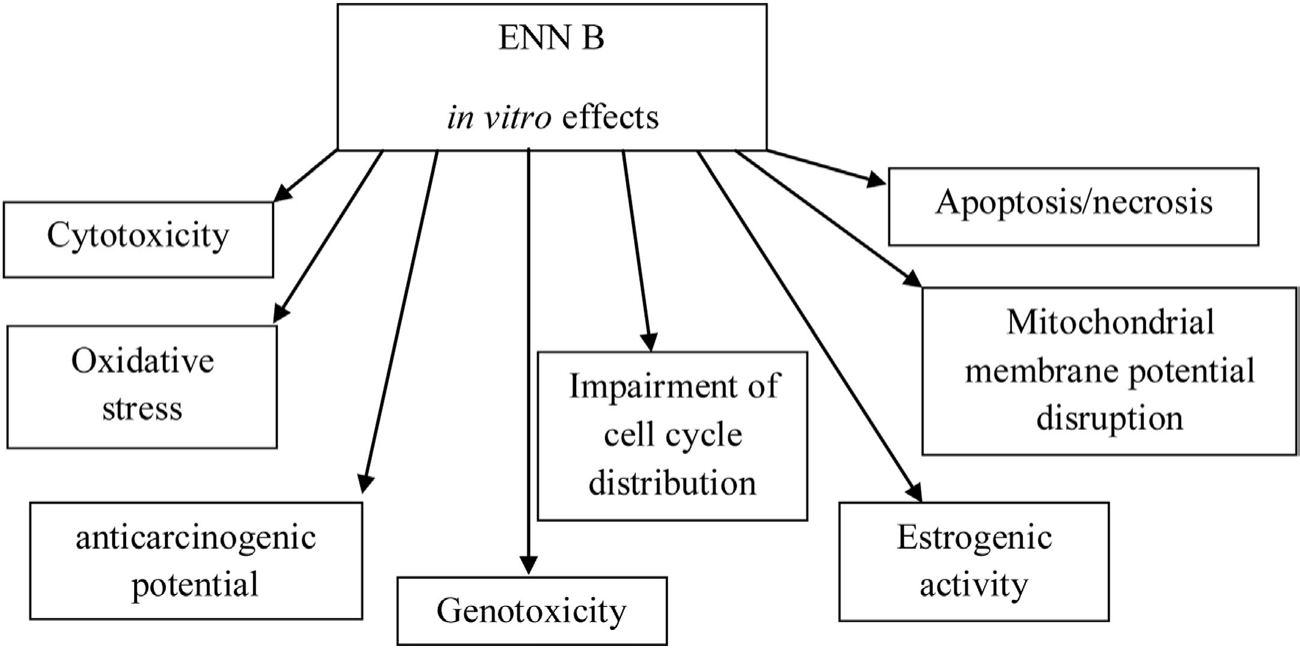
Schema showing *in vitro* effects of enniatin B (ENN B).

Comparing to *in vitro* studies, the number of studies *in vivo* is very low. *In vitro* cytotoxicity studies have been carried out for individual ENN B as well as for mixtures of ENNs, since mycotoxins, either from the same or from different fungal species, occur simultaneously in plant and food products (12). A scheme of *in vitro* studies on ENN B is shown in Figure 3.

### *In Vitro* Cytotoxicity

#### Cytotoxicity Studies of Individual ENN B

Different cell lines and assays have been chosen to determine ENN B cytotoxicity. Table 2 collects the cytotoxic activity studies performed in several cell lines exposed to ENN B tested individually and in complex with other ENNs (ENN A, A1, and B1) according to the type of cells, the toxicity endpoint, and the time of exposure. Data from literature show that human colon intestinal Caco-2 cells have been the most studied cell line when ENN B is applied alone (not in complex mixture), followed by HepG2 and CHO-K1 cells (Table 2). Exposure time goes from 3 to 72 h and the ENN B IC_50_ values ranged in: (i) Caco-2 cells from 1.4 to >30 μM; (ii) HepG2 cells, from 0.9 to 435.9 µM; and (iii) CHO-K1 cells from 2.80 ± 0.16 to 11 µM. The lowest value obtained was 0.50 of ED_50_ in HepG2 cells, suggesting higher sensitivity of this type of cells to ENN B. HT-29, MRC-5, and V7 cell lines were also studied with different ENNs individually and IC_50_ concentrations ranged from 1.4 to 16.8 µM for HT-29, from 0.6 to 9.8 µM for MRC-5, and from 2.5 ± 0.4 to 43 ± 20 µM for V7 cells, respectively.

**Table 2:** *In vitro* cytotoxicity studies on enniatin B determined by different toxicity endpoint, time of exposure and cell types.

Cell lines	Cell types	Parameter	Exposure time (h)	IC_50_ (μM)	Reference
A427	Human lung cancer	MTT	72	ENN_mix_: 1.61 ± 0.14	Dornetshuber et al. (33)
A549				ENN_mix_: 4.08 ± 1.04	

Balb 3T3	Mouse embryo fibroblast	ATP production	24	ED_50_: 8.4 ± 0.76	Jonsson et al. (13)
		BrdU ELISA		ED_50_: 4.24 ± 0.06	
		Apoptosis induction		ED_50_: 11	

Caco-2	Human colon adenocarcinoma	NR	3, 24	10.0 ± 3.8–2.1 ± 0.4	Ivanova et al. (10)
			24, 48, 72	>15–1.4 ± 0.2	Prosperini et al. (16)
			24, 48	>30	Meca et al. (44)
			24, 48	No IC_50_ value obtained	Meca et al. (64)
			72	ENN_mix_: 1.99 ± 0.09	Dornetshuber et al. (33)
			24, 48, 72	>15–11.7 ± 2.4	Prosperini et al. (31)

		WST-1	24	6.3	Vejdovszky et al. (65)

CHO-K1	Chinese hamster ovary	MTT	24, 48, 72	11.0 ± 2.65–2.80 ± 0.16	Lu et al. (30)
			24	11.0 ± 2.65	Lombardi et al. (66)

C6	Rat glioma	MTT	3, 24	ENN_mix_: 2.5–10	Wätjen et al. (34)

GLC-4	Breast adenocarcinoma	MTT	72	ENN_mix_: 2.40 ± 1.53	Dornetshuber et al. (33, 61)

GLC-4/adr	MVP-overexpressing subline	MTT	72	ENN_mix_: 1.41 ± 0.83	Dornetshuber et al. (61)

GBL1	Human Glioblastoma	MTT	72	ENN_mix_: 2.65 ± 0.30	Dornetshuber et al. (33)
GBL2				ENN_mix_: 2.29 ± 0.05	
GBL3				ENN_mix_: 2.55 ± 0.14	
GBL4				ENN_mix_: 2.33 ± 0.30	

H-4IIE	Rat hepatocarcinoma	MTT	3, 24	ENN A1, B and B1: 1–1.5	Wätjen et al. (34)

Hep G2	Human hepatocarcinoma	MTT	24, 48	No IC_50_ value obtained	Meca et al. (64)
			3, 24	ENN A1, B and B1: 10	Wätjen et al. (34)

		Alamar Blue BrdU	24	206.7–435.9 0.9–1.1	Ivanova et al. (18)

		ATP production	24	ED_50_: 2.9 ± 0.7	Jonsson et al. (13)
		
		BrdU ELISA	ED_50_: 0.50 ± 0.09
		
		Qaudroprobe multiparametric liver toxicity assay^a^	24, 72	0.9^b^	Svingen et al. (27)

HL60	Promyelocityc leukemia carcinoma	MTT	72	ENN_mix_: 1.74 ± 0.20	Dornetshuber et al. (33, 61)

HL60/vinc	ABCB1-overexpressing subline	MTT	72	ENN_mix_: 2.40 ± 0.14	Dornetshuber et al. (61)

HL60/adr	ABCC1-overexpressing subline	MTT	72	ENN_mix_: 2.1 ± 0.12	Dornetshuber et al. (61)

HT-29	Human colon adenocarcinoma	MTT	24, 48	2.8 ± 0.9	Meca et al. (64)

HUVEC	Human endothelial	MTT	72	ENN_mix_: 7.89 ± 0.21	Dornetshuber et al. (33)

KB-3-1	Epidermal carcinoma	MTT	72	ENN_mix_: 1.95–0.12	Dornetshuber et al. (33, 61)

KBC-1	ABCB1-overexpressing subline	MTT	72	ENN_mix_: 1.77 ± 0.04	Dornetshuber et al. (61)

MDA-MB-231	Alveolar epithelial	MTT	72	ENN_mix_: 2.36 ± 1.57	Dornetshuber et al. (33, 61)

MDA-MB-231/adr	ABCG2-overexpressing subline	MTT	72	ENN_mix_: 3.18 ± 1.70	Dornetshuber et al. (61)

MGC	Human glioblastoma	MTT	72	ENN_mix_: 3.04 ± 0.58	Dornetshuber et al. (33)

MRC-5	Fibroblast-like fetal lung	Alamar Blue BrdU	24	1.9–9.8	Ivanova et al. (18)
				1.9–3.6	
OS 9	Human osteosarcoma	MTT	72	ENN_mix_: 3.55 ± 0.77	Dornetshuber et al. (33)
OS 10				ENN_mix_: 2.10 ± 0.15	

Porcine kidney cells-15	Porcine Kidney	MTT	72	ENN_mix_: 41	Uhlig et al. (67)

RAW 264.7	Murine macrophage	Alamar blue	24	2.6	Gammelsrud et al. (35)
		NR		4.7	

SAOS	Human osteosarcoma	MTT	72	ENN_mix_: 2.13 ± 0.07	Dornetshuber et al. (33)

SW1537	Small cell lung carcinoma	MTT	72	ENN_mix_: 2.16 ± 0.12	Dornetshuber et al. (61)

SW1537/2R160	ABCB1-overexpressing subline	MTT	72	ENN_mix_: 2.69 ± 0.60	Dornetshuber et al. (61)

SW480	Small cell lung carcinoma	MTT	72	ENN_mix_: 4.00 ± 1.12	Dornetshuber et al. (33)

T98-G	Human glioblastoma	MTT	72	ENN_mix_: >10	Dornetshuber et al. (33)

U373	Human glioblastoma	MTT	72	ENN_mix_: 4.88 ± 0.09	Dornetshuber et al. (33)

U2-OS	Osteosarcoma	MTT	72	ENN_mix_: 1.77 ± 0.24	Dornetshuber et al. (33)

V79	Chinese hamster fibroblast	NR	48	4.4	Dornetshuber et al. (68)

		Alamar blue	24–48	34 ± 20–2.5 ± 0.4	Föllmann et al. (69)
		NR		36 ± 16–4 ± 1.5 43 ± 20–3.9 ± 3.9	
		Protein content (BCA)			

VM8	Melanoma	MTT	72	ENN_mix_: 3.19 ± 0.85	Dornetshuber et al. (33)
VM18				ENN_mix_: 2.67 ± 0.08	
VM22				ENN_mix_: 1.75 ± 0.15	
VM33				ENN_mix_: 9.65 ± 0.13	
VM25				ENN_mix_: 2.72 ± 0.11	

VL8	Human lung cancer	MTT	72	ENN_mix_: >10	Dornetshuber et al. (33)

WI-38	Embryonic fibroblast	MTT	72	ENN_mix_: >10	Dornetshuber et al. (33)

*IC_50_ = inhibitory concentration at which 50% of enzyme activity is inhibited; ED_50_ = effective dose at which 50% of cells are affected*.

*^a^Usingfour fluorophore probes, six cytotoxicity-associated parameters were analyzed simultaneously: nuclear count and size, plasma membrane integrity, lysosomal activity, mitochondrial membrane potential, and mitochondrial area*.

*^b^The concentration that deviated significantly from the control cell responses*.

#### Cytotoxicity Studies of Combined Mycotoxins Including ENN B

There are several studies involving mixtures of ENNs including ENN B, as shown in Table 2; some of these ENNs mixtures do not indicate exactly the percentage of each ENN. Review of the literature showed that the lowest IC_50_ value obtained with mixture of ENNs was for MVP-overexpressing subline cells (GLC-4/adr) with 1.41 ± 0.20 µM at 72 h and, the highest was obtained in porcine kidney cells (PK-15) with 41 µM at 72 h, followed by IC_50_ > 10 µM at 72 h for human lung cancer cells (VL8), embryonic fibroblast cells (WI-38), and human glioblastoma cells (T98-G).

Studies about cytotoxicity effects of ENN B in combination with other ENNs, and ENN B in combination with other mycotoxins, are collected in Table 3. ENN B was tested in combination with ENN A, A1, and B1 by Prosperini et al. (31) in Caco-2 cells and by Lu et al. (30) in CHO-K1 cells. Both studies aimed to investigate the type of interaction that occurs when ENNs appear in combination such as: synergism, antagonism, or additive effect by using isobologram method (70). The analysis was performed by testing binary and ternary combination in CHO-K1 and binary, ternary, and quaternary combination in Caco-2 cells. Prosperini et al. (31) reported a reduction of Caco-2 cell viability (%) in a dose-dependent manner for binary mixture tested in the following increasing order: ENN A + ENN A1 (48%) = ENN A1 + ENN B1 (47%) > ENN A1 + ENN B (35%) = ENN A + ENN B (33%) = ENN A + ENN B1 (32%) > ENN B + ENN B1 (26%). Similarly, tertiary and quaternary mixtures reduced cell viability in a dose-dependent manner with a reduction in viability of approximately 40%. Generally, additive cytotoxic effect was observed for all combinations; however, synergistic effects were observed for the following mixtures: ENN B + ENN A1, ENN B1 + ENN A1, and ENN A + ENN A1 + ENN B and, a moderate antagonism was produced by ENN B + ENN B1 combination.

**Table 3:** Combinedeffect of ENN B tested in combination with other mycotoxins by *in vitro* methods.

Cell line	Mycotoxin combination	Effect	Interaction (in the combination)	Reference
Caco-2	ENN A + A1	Cytotoxicity	Add	Prosperini et al. (31)
	ENN A + B			
	ENN A + B1			
	ENN B + B1			
	ENN A + A1 + B1			
	ENN A + B + B1			
	ENN A1 + B + B1			
	ENN A + A1 + B + B1			
	ENN A1 + B1		Syn	
	ENN A1 + B			
	ENN A + A1 + B			
	ENN B + B1		Ant	

	ENN B + ZEA	Cytotoxicity	Ant	Vejdovszky et al. (65)
	ENN B + DON			
	ENN B + NIV		Strong	
			Antagonism	

	ENN B + AOH		Syn	Fernandez-Blanco et al. (15)
	ENN B + DON		Add/Syn	
	ENN B + AOH + DON		Add/Syn	

CHO-K1	ENN A + B1	Cytotoxicity	Add	Lu et al. (30)
	ENN A1 + B			
	ENN B + B1			
	ENN A + A1		Syn	
	ENN A + B			
	ENN A1 + B1			
	ENN A + A1 + B			
	ENN A + B + B1			
	ENN A + A1 + B1		Ant	
	ENN A1 + B + B1			

Human hematopoietic progenitors	BEA + ENN B	Myelotoxicity	Add	Ficheux et al. (71)

*AOH, alternariol; DON, deoxynivalenol; ZEA, zearalenone; NIV, nivalenol; ENN, Enniatin*.

Lu et al. (30) found that the binary combinations ENNs A + B1, ENNs A1 + B, and ENNs B + B1 showed additive effects with all concentrations tested in CHO-K1 cells. Synergistic effect of combined ENNs A + A1, A + B, A1 + B1, A + A1 + B, A + A1 + B1, A + B + B1, and A1 + B + B1 at higher concentrations occurred. Synergism effect was observed at higher concentrations with binary and tertiary combinations of ENN A, while antagonism effects were obtained at lower concentrations for ENNs A + A1 + B1 and ENNs A1 + B + B1.

In addition, studies about the combination of ENNs with other mycotoxins have been carried out (15, 65, 71). Briefly, ENN B, tested with beauvericin, had additive cytotoxic effect on Human hematopoietic progenitors (71). Binary mixtures of ENN B with ZEA, DON, and nivalenol showed antagonistic and strong antagonistic effects on Caco-2 cell viability (65). As Fernandez-Blanco et al. (15) reported, mixtures of ENN B + DON and ENN B + Alternariol (AOH) were found to be synergic, depending on the concentrations tested.

### Oxidative Stress

One of the key players in the production of oxidative stress is reactive oxygen species (ROS). Moreover, intracellular ROS generation in the hydrophobic compartment of a cell can induce lipid peroxidation (LPO).^8^ ROS generation and LPO have been observed in mammalian cells exposed to ENN B (10, 16). In addition, Ivanova et al. (10) found that ENN B-induced ROS production after 3 h exposure to ENN B in Caco-2 cells that were generated downstream the ENN B-induced cytotoxic events by the mitochondria. On the contrary, Dornetshuber (68) demonstrated that genotoxic potential and cytotoxicity of ENNs is independent of ROS generation. Further research is needed in this area.

### Impairment of Cell Cycle Distribution

Cell cycle is the entire process by which a cell undergoes cell division. Cell cycle phases are: the G1 phase, where cells are preparing for DNA, RNA, and protein synthesis, the S phase where DNA is synthesized, the G2 phase, where cells are preparing for mitosis, and finally the M phase (mitosis) where two daughter cells are generated. Cells can remain in a quiescent phase (G0 phase) and they need growth factors to enter the G1 phase.

It has been demonstrated that mycotoxins can disturb the normal cell cycle distribution due to their anti-proliferative effects on several cell types, with an accumulation of cells in one or more phases of the cell cycle (14)^9^. Juan-García et al. (72) observed that in HepG2, ENN B provoked a higher percentage of cells arrested at G0/G1 at 48 h than ENN A. A similar behavior was reported by Gammelsrud et al. (35) on murine monocytes macrophage RAW 267.4 cells: an increase in percentage of cells in G0/G1 phase when treated during 24 h with ENN B (from 1.25 to 10 µM). In general, cell accumulation during cell cycle in any phase after a treatment indicates an anti-proliferative activity of the compound or compounds used. The same behavior was observed in several human cancer cell lines by Dornetshuber et al. (33).

In Caco-2 cells, as described by Prosperini et al. (16), ENN B (3 µM) induced an arrest of the cell cycle in G2/M phase after 72 h of exposure with a significant increase in G2/M cell number compared to the control. A decrease of the S phase cell population and an increase in SubG0/G1 were also observed.

A noticeable increase of cells in the G2/M phase in Caco-2 cells after ENN B treatment was also observed by Ivanova et al. (10) confirming the impairment of mitosis. This type of arrest has been described as a possible consequence of external stimuli leading to apoptosis by activation of the caspase pathway or to non-apoptotic mitotic death.

### Apoptosis/Necrosis

Enniatin B alone and in mixture with other ENNs induced apoptosis in several cell lines with nuclear fragmentation and apoptotic body formation (13, 14, 16, 33–35, 68, 69, 72). Moreover, the necrotic cell death has been also reported (10, 16, 35). Apoptotic effect and necrotic^10^ pathway was observed in Caco-2 cells after 48 h of exposure to ENN B (10, 16).

Juan-García et al. (72) found that both ENN B and ENN A (1.5 and 3.0 µM) caused apoptosis in HepG2 cells, after 48 h of exposure, identifying ENN B more toxic than ENN A. Necrotic pathway was not observed. Similar results were obtained in mouse embryo fibroblast (Balb 3T3) cells (from 11 to 45 µM) (13), in murine monocyte (RAW 267.4) macrophages (35) and in human adrenocortical carcinoma cell line H295R (36).

### MMP Disruption

Mitochondria have been recognized for their role in mediating physiological processes and their involvement in signal transduction and regulation of cell proliferation and differentiation. They are involved also in cell death regulation, i.e., necrosis and apoptosis. Due to this role, mitochondria are vulnerable to the toxic effects of xenobiotics that interfere in cellular energy production. Apoptosis and necrosis induced cell death by cytotoxic agents involve similar metabolic disturbances and above all, mitochondrial permeability transition (MPT). Mitochondrial events of apoptosis and necrosis involve opening of a pore in the inner mitochondrial membrane, referred as mitochondrial PTP (MPTP) and the consequent dissipation of membrane potential (73). The dissipation of the MMP results from the unequal distribution of ions (mainly protons) on the inner mitochondrial membrane. The MMP disruption suggests that the proton-moving force and/or the inner membrane permeability has been affected during cell damage. The dissipation of MMP is a general feature of both cell death types (16).

Measurements of MMP are carried out by using lipophylic dyes, which pass through cell membranes and accumulate according to their charge. The alteration of fluorescent intensity can be determined by flow cytometry. Among these dyes, the tetramethylrhodamine methyl ester (TMRM), coupled with the carbocyanine monomer nucleic acid (To-Pro-3), has been used to determine the mitochondrial starting depolarization and cells progressing to death through apoptosis (72).

The disruption of MMP has been demonstrated in KB-3-1 cells exposed to ENNs mixture (33), and to ENN B in Caco-2 and HepG2 cells (10, 16, 72). The exact mechanism by which pro-oxidant mycotoxins induced pore opening is still not fully understood. At least two molecular sites of the complex contribute to this effect. The first site is a redox sensitive membrane dithiol group that can be oxidized by ROS (produced by mycotoxins), the second one remains undetermined.

Mitochondrial membrane potential was measured also by Svingen et al. (27) by the Hep-G2 quadroprobe multi-parametric liver toxicity assay. The strongest effect was seen for plasma membrane integrity, with concomitant effects on mitochondrial area/mass and mitochondrial potential, confirming the involvement of mitochondria in ENNs toxicity (11).

### Estrogenic Activity

Recently, an investigation to evaluate possible endocrine disruptor effects^11^ of ENN B was conducted by Kalayou et al. (36) demonstrating that in the human adrenocortical carcinoma cell line H295R, ENN B (10 µM) was able to reduce progesterone, testosterone, and cortisol production at a non-cytotoxic concentration. Higher concentrations (>10 μM) reduced both estradiol and testosterone levels in Leydig cells (36). Additional research should be conducted using ovarian steroid producing cells such as granulosa cells.

### Genotoxicity

Genotoxicity^12^ seems to be not involved in ENN B induction of cell death. As reported by Follmann et al. (69), ENN B, despite its high cytotoxic potential, did not induce any DNA damage by the alkaline Comet Assay in V79 cells after concentrations ranging from 1 to 100 µM. Results are in accordance with those obtained by Gammelsrud et al. (35) and Prosperini et al. (16) in Caco-2 cells.

## Emerging Findings of ENN B

Some researchers are underlying the anticancer potential of ENN B (33, 34, 61, 68). Apoptosis with the involvement of p53 and p21 genes was found by Dornetshuber et al. (33), which tested a mixture of ENNs against several human cancer cells, promoting ENNs as anticancer drugs, according also to Wätjen et al. (34) and Dornetshuber-Fleiss et al. (37). In these surveys, ENNs caused caspase 3/7 activation in hepatoma H4IIE cells and caspase-7 activation in the KB-3-1 cell line, respectively, as well as nuclear fragmentation.

Enniatin B is capable of resisting expulsion by the ABC transporters, and also naturally targets tumor cells more specifically than other chemotherapeutic agents. The action is synergic with the clinically approved multi-kinase inhibitor sorafenib (Sora) showing profound synergistic *in vitro* and *in vivo* anticancer effects against cervical cancer (37).

## Concluding Remarks

Mycotoxins constitute a serious health concern both for animals and for humans, besides economic problems. Productive and nutritive values of food and feed can be compromised by mold and mycotoxin contamination, and toxicological risk derived by ingestion is constantly under Authorities control. Regarding ENNs, a risk assessment is still not available, despite its clear toxicity *in vitro* and its presence in food and feed.

Indeed, several *in vitro* and *in vivo* studies have revealed that ENN B interacts with primary target molecules, induce signaling pathways and effector mechanisms, affects the biological response of cell defenses, promotes cell damage, produces potential interactions between food contaminants (particularly other mycotoxins) leading to abnormally high response, and other molecular events underlying ENN B toxicity. Nevertheless, regulatory limits have not yet been defined, due to a lack of complete toxicity data.

However, in the last decade, novel findings about a potential therapeutic action of ENNB have been proven. These promising findings introduce a new aspect of this toxic compound. Future research focused on elucidating the toxic mechanism of ENN B as well as its anticancer activity could better clarify the real potential of ENN B. These research findings could contribute to establish emerging therapeutic strategy to chronic health problems.

This review wanted to collect all available data regarding toxicological aspect and emerging findings on ENN B in order to underlying the need to continue to study toxic/emerging effects of this compound to finally protect and improve both animal and human well-being.

## Author Contributions

AP is the first author (40%). HB (5%), M-JL (10%), FC (10%), TC (5%), LS (10%), MP (10%), and AL (10%).

## Conflict of Interest Statement

The authors declare that the research was conducted in the absence of any commercial or financial relationships that could be construed as a potential conflict of interest.

## Footnotes

^1^*Fusarium* is a large and diverse genus of filamentous fungi of great agricultural and economic importance, containing many plant pathogens and mycotoxin producers.

^2^Mycotoxins are secondary metabolites produced by microfungi that are capable of causing disease and death in humans and other animals.

^3^Ionophores are molecules that facilitate ion passage in or out of cell membranes.

^4^ACAT is an intracellular enzyme located in the endoplasmic reticulum that transfer fatty acyl groups from one molecule to another.

^5^Oxidative stress is defined as a disturbance in the balance between the production of reactive oxygen species (free radicals) and antioxidant defenses.

^6^Yeast multidrug resistance protein that belongs to the family of ABC transporters. Pdr5p has been shown to confer resistance to a wide range of compounds and metal ions.

^7^The ABC transporter superfamily is the largest transporter gene family. These proteins translocate a wide variety of substrates including sugars, amino acids, metal ions, peptides, and proteins, and a large number of hydrophobic compounds and metabolites across extra- and intracellular membranes.

^8^Lipid peroxidation can be described generally as a process under which oxidants such as free radicals attack lipids containing carbon-carbon double bond(s), especially polyunsaturated fatty acids.

^9^The natural cell cycle includes a number of checkpoints that allow the cell to determine whether to proceed with division or stop. These halts can also be induced by external factors like chemicals. Research on cell cycle arrest provides important information about how cells regulate themselves and what happens when these processes go wrong.

^10^Two different ways of cells can die are apoptosis or necrosis. Apoptosis is described as an active, programmed process of autonomous cellular dismantling that avoids eliciting inflammation. Necrosis has been characterized as passive, accidental cell death resulting from environmental perturbations with uncontrolled release of inflammatory cellular contents.

^11^Chemicals that mimic or antagonize the *in vitro* and/or *in vivo* actions of naturally occurring estrogens are typically defined as having estrogenic activity or anti-estrogenic activity. Effects on estrogen signaling represent the most common and best studied endocrine disruptor activity.

^12^Genotoxicity is defined as the destructive effect on a cell’s genetic material (DNA and RNA) affecting its integrity.

## References

[1] Logrieco A Moretti A Mule G Paciolla C Ritieni A Advances on the toxicity of the cereal contaminant *Fusarium* esadepsipeptides. Cereal Res Commun (2008) 36:303–13. 10.1556/CRC.36.2008.Suppl.B.28

[2] Juan C Mañes J Raiola A Ritieni A. Evaluation of beauvericin and enniatins in Italian cereal products and multicereal food by liquid chromatography coupled to triple quadrupole mass spectrometry. Food Chem (2013) 140(4):755–62. 10.1016/j.foodchem.2012.08.021 23692763

[3] Covarelli L Beccari G Prodi A Generotti S Etruschi F Juan C *Fusarium* species, chemotype characterisation and trichothecene contamination of durum and soft wheat in an area of central Italy. J Sci Food Agric (2015) 95(3):540–51. 10.1002/jsfa.6772 24909776

[4] García-Moraleja A Font G Mañes J Ferrer E. Analysis of mycotoxins in coffee and risk assessment in Spanish adolescents and adults. Food Chem Toxicol (2015) 86:225–33. 10.1016/j.fct.2015.10.014 26514696

[5] Quiles JM Saladino F Mañes J Fernández-Franzón M Meca G. Occurrence of mycotoxins in refrigerated pizza dough and risk assessment of exposure for the Spanish population. Food Chem Toxicol (2016) 94:19–24. 10.1016/j.fct.2016.05.011 27222027

[6] Tolosa J Font G Mañes J Ferrer E. Mitigation of enniatins in edible fish tissues by thermal processes and identification of degradation products. Food Chem Toxicol (2017) 101:67–74. 10.1016/j.fct.2016.12.039 28043835

[7] Zinedine A Fernández-Franzón M Mañes J Manyes L. Multi-mycotoxin contamination of couscous semolina commercialized in Morocco. Food Chem (2017) 214:440–6. 10.1016/j.foodchem.2016.07.098 27507496

[8] de Nijs M van den Top H de Stoppelaar J Lopez P Mol H. Fate of enniatins and deoxynivalenol during pasta cooking. Food Chem (2016) 213:763–7. 10.1016/j.foodchem.2016.07.024 27451245

[9] Luz C Saladino F Luciano FB Mañes J Meca G. Occurrence, toxicity, bioaccessibility and mitigation strategies of beauvericin, a minor *Fusarium* mycotoxin. Food Chem Toxicol (2017) 107(Pt A):430–9. 10.1016/j.fct.2017.07.032 28720287

[10] Ivanova L Egge-Jacobsen WM Solhaug A Thoen E Faeste CK. Lysosomes as a possible target of enniatin B-induced toxicity in Caco-2 cells. Chem Res Toxicol (2012) 25(8):1662–74. 10.1021/tx300114x 22731695

[11] Tonshin AA Teplova VV Andersson MA Salkinoja-Salonen MS. The *Fusarium* mycotoxins enniatins and beauvericin cause mitochondrial dysfunction by affecting the mitochondrial volume regulation, oxidative phosphorylation and ion homeostasis. Toxicology (2010) 276(1):49–57. 10.1016/j.tox.2010.07.001 20621153

[12] Jestoi M. Emerging *Fusarium*-mycotoxins fusaproliferin, beauvericin, enniatins, and moniliformin: a review. Crit Rev Food Sci Nutr (2008) 48(1):21–49. 10.1080/10408390601062021 18274964

[13] Jonsson M Jestoi M Anthoni M Welling A Loivamaa I Hallikainen V *Fusarium* mycotoxin enniatin B: cytotoxic effects and changes in gene expression profile. Toxicol In Vitro (2016) 34:309–20. 10.1016/j.tiv.2016.04.017 27163883

[14] Juan-García A Manyes L Ruiz MJ Font G. Involvement of enniatins-induced cytotoxicity in human HepG2 cells. Toxicol Lett (2013) 218(2):166–73. 10.1016/j.toxlet.2013.01.014 23370383

[15] Fernandez-Blanco C Font G Ruiz MJ. Interaction effects of enniatin B, deoxinivalenol and alternariol in Caco-2 cells. Toxicol Lett (2016) 241:38–48. 10.1016/j.toxlet.2015.11.005 26581633

[16] Prosperini A Juan-García A Font G Ruiz MJ. Reactive oxygen species involvement in apoptosis and mitochondrial damage in Caco-2 cells induced by enniatins A, A^1^, B and B1. Toxicol Lett (2013) 222(1):36–44. 10.1016/j.toxlet.2013.07.009 23867914

[17] Behm C Degen GH Föllmann W. The *Fusarium* toxin enniatin B exerts no genotoxic activity, but pronounced cytotoxicity in vitro. Mol Nutr Food Res (2009) 53(4):423–30. 10.1002/mnfr.200800183 19360736

[18] Ivanova L Skjerve E Eriksen GS Uhlig S. Cytotoxicity of enniatins A, A1, B, B1, B2 and B3 from *Fusarium avenaceum*. Toxicon (2006) 47(8):868–76. 10.1016/j.toxicon.2006.02.012 16730043

[19] McKee TC Bokesch HR McCormick JL Rashid MA Spielvogel D Gustafson KR Isolation and characterization of new anti-HIV and cytotoxic leads from plants, marine, and microbial organisms. J Nat Prod (1997) 60(5):431–8. 10.1021/np970031g 9170286

[20] Juan C Manyes L Font G Juan-García A. Evaluation of immunologic effect of enniatin A and quantitative determination in feces, urine and serum on treated Wistar rats. Toxicon (2014) 87:45–53. 10.1016/j.toxicon.2014.05.005 24857789

[21] Manyes L Escrivá L Serrano AB Rodríguez-Carrasco Y Tolosa J Meca G A preliminary study in Wistar rats with enniatin A contaminated feed. Toxicol Mech Methods (2014) 24(3):179–90. 10.3109/15376516.2013.876135 24329503

[22] Rodríguez-Carrasco Y Heilos D Richter L Süssmuth RD Heffeter P Sulyok M Mouse tissue distribution and persistence of the food-born fusariotoxins enniatin B and beauvericin. Toxicol Lett (2016) 247:35–44. 10.1016/j.toxlet.2016.02.008 26892719 PMC5850989

[23] Escrivá L Font G Manyes L. Quantitation of enniatins in biological samples of Wistar rats after oral administration by LC-MS/MS. Toxicol Mech Methods (2015) 25(7):552–8. 10.3109/15376516.2015.1061083 26228087

[24] Benford D Ceccatelli S Cottrill B Dinovi M Dogliotti E Edler L Scientific opinion on the risks to human and animal health related to the presence of beauvericin and enniatins in food and feed. EFSA J (2014) 12(8):3802 10.2903/j.efsa.2014.3802

[25] Dagnac T Latorre A Fernández Lorenzo B Llompart M. Validation and application of a liquid chromatography-tandem mass spectrometry based method for the assessment of the co-occurrence of mycotoxins in maize silages from dairy farms in NW Spain. Food Addit Contam Part A Chem Anal Control Expo Risk Assess (2016) 33(12):1850–63. 10.1080/19440049.2016.1243806 27707355

[26] Decleer M Rajkovic A Sas B Madder A De Saeger S. Development and validation of ultra-high-performance liquid chromatography-tandem mass spectrometry methods for the simultaneous determination of beauvericin, enniatins (A, A1, B, B1) and cereulide in maize, wheat, pasta and rice. J Chromatogr A (2016) 1472:35–43. 10.1016/j.chroma.2016.10.003 27776774

[27] Svingen T Lund Hansen N Taxvig C Vinggaard AM Jensen U Have Rasmussen P. Enniatin B and beauvericin are common in Danish cereals and show high hepatotoxicity on a high-content imaging platform. Environ Toxicol (2017) 32(5):1658–64. 10.1002/tox.22367 27628925

[28] Uhlig S Jestoi M Parikka P *Fusarium avenaceum* – the North European situation. Int J Food Microbiol (2007) 119(1–2):17–24. 10.1016/j.ijfoodmicro.2007.07.021 17884217

[29] Habler K Gotthardt M Schüler J Rychlik M Multi-mycotoxin stable isotope dilution LC–MS/MS method for *Fusarium* toxins in beer. Food Chem (2017) 218:447–54. 10.1016/j.foodchem.2016.09.100 27719934

[30] Lu H Fernández-Franzón M Font G Ruiz MJ. Toxicity evaluation of individual and mixed enniatins using an in vitro method with CHO-K1 cells. Toxicol In Vitro (2013) 27(2):672–80. 10.1016/j.tiv.2012.11.009 23168488

[31] Prosperini A Font G Ruiz MJ. Interaction effects of *Fusarium* enniatins (A, A1, B and B1) combinations on in vitro cytotoxicity of Caco-2 cells. Toxicol In Vitro (2014) 28(1):88–94. 10.1016/j.tiv.2013.06.021 23850737

[32] Tomoda H Huang XH Cao J Nishida H Nagao R Okuda S Inhibition of acyl-CoA: cholesterol acyltransferase activity by cyclodepsipeptide antibiotics. J Antibiot (Tokyo) (1992) 45(10):1626–32. 10.7164/antibiotics.45.1626 1473990

[33] Dornetshuber R Heffeter P Kamyar MR Peterbauer T Berger W Lemmens-Gruber R. Enniatin exerts p53-dependent cytostatic and p53-independent cytotoxic activities against human cancer cells. Chem Res Toxicol (2007) 20(3):465–73. 10.1021/tx600259t 17326668

[34] Wätjen W Debbab A Hohlfeld A Chovolou Y Kampkötter A Edrada RA Enniatins A1, B and B1 from an endophytic strain of *Fusarium tricinctum* induce apoptotic cell death in H4IIE hepatoma cells accompanied by inhibition of ERK phosphorylation. Mol Nutr Food Res (2009) 53(4):431–40. 10.1002/mnfr.200700428 19065580

[35] Gammelsrud A Solhaug A Dendele B Sandberg WJ Ivanova L Kocbach Bolling A Enniatin B-induced cell death and inflammatory responses in RAW 267.4 murine macrophages. Toxicol Appl Pharmacol (2012) 261(1):74–87. 10.1016/j.taap.2012.03.014 22483798

[36] Kalayou S Ndossi D Frizzell C Groseth PK Connolly L Sørlie M An investigation of the endocrine disrupting potential of enniatin B using in vitro bioassays. Toxicol Lett (2015) 233(2):84–94. 10.1016/j.toxlet.2015.01.014 25625232

[37] Dornetshuber-Fleiss R Heilos D Mohr T Richter L Süssmuth RD Zlesak M The naturally born fusariotoxin enniatin B and sorafenib exert synergistic activity against cervical cancer in vitro and in vivo. Biochem Pharmacol (2015) 93(3):318–31. 10.1016/j.bcp.2014.12.013 25557295 PMC4379350

[38] Grove JF Pople M The insecticidal activity of beauvericin and the enniatin complex. Mycopathologia (1980) 70(2):103–5. 10.1007/BF00443075

[39] Strongman DB Strunz GM Giguère P Yu C-M Calhoun L Enniatins from *Fusarium avenaceum* isolated from balsam fir foliage and their toxicity to spruce budworm larvae, *Choristoneura fumiferana* (Clem.) (Lepidoptera: Tortricidae). J Chem Ecol (1988) 14(3):753–64. 10.1007/BF01018770 24276128

[40] Ciancio A Observations on the nematicidal properties of some mycotoxins. Fundam Appl Nematol (1995) 18(5):451–4.

[41] Pleiss U Turberg A Harder A Londershausen M Jeschke P Boheim G Synthesis of a radiolabeled enniatin cyclodepsipeptide [3H-methyl]JES 1798. J Labelled Comp Radiopharm (1996) 38(7):651–9. 10.1002/(SICI)1099-1344(199607)38:7<651::AID-JLCR881>3.0.CO;2-S

[42] Pohanka A Capieau K Broberg A Stenlid J Stenström E Kenne L. Enniatins of *Fusarium* sp. strain F31 and their inhibition of *Botrytis cinerea* spore germination. J Nat Prod (2004) 67(5):851–7. 10.1021/np0340448 15165149

[43] Mulè G D’Ambrosio A Logrieco A Bottalico A Toxicity of mycotoxins of *Fusarium sambucinum* for feeding in *Galleria mellonella*. Entomol Exp Appl (1992) 62(1):17–22. 10.1111/j.1570-7458.1992.tb00636.x

[44] Meca G Sospedra I Valero MA Mañes J Font G Ruiz MJ. Antibacterial activity of the enniatin B, produced by *Fusarium tricinctum* in liquid culture, and cytotoxic effects on Caco-2 cells. Toxicol Mech Methods (2011) 21(7):503–12. 10.3109/15376516.2011.556202 21417626

[45] Vesonder RF Golinski P Metabolites of *Fusarium*. In: Chelkowski J, editor. Fusarium: Mycotoxins, Taxonomy and Pathogenicity. Amsterdam, The Netherlands: Elsevier (1989). p. 1–39.

[46] Nilanonta C Isaka M Chanphen R Thong-orn N Tanticharoen M Thebtaranonth Y Unusual enniatins produced by the insect pathogenic fungus *Verticillium hemipterigenum*: isolation and studies on precursor-directed biosynthesis. Tetrahedron (2003) 59(7):1015–20. 10.1016/S0040-4020(02)01631-9

[47] Supothina S Isaka M Kirtikara K Tanticharoen M Thebtaranonth Y. Enniatin production by the entomopathogenic fungus *Verticillium hemipterigenum* BCC 1449. J Antibiot (Tokyo) (2004) 57(11):732–8. 10.7164/antibiotics.57.732 15712668

[48] Meca G Soriano JM Gaspari A Ritieni A Moretti A Mañes J. Antifungal effects of the bioactive compounds enniatins A, A(1), B, B(1). Toxicon (2010) 56(3):480–5. 10.1016/j.toxicon.2010.04.013 20417654

[49] Herrmann M Zocher R Haese A. Enniatin production by *Fusarium* strains and its effect on potato tuber tissue. Appl Environ Microbiol (1996) 62(2):393–8. 16535227 10.1128/aem.62.2.393-398.1996 PMC1388765

[50] Hershenhorn J Park SH Stierle A Strobel GA *Fusarium avenaceum* as a novel pathogen of spotted knapweed and its phytotoxins, acetamido-butenolide and enniatin B. Plant Sci (1992) 86(2):155–60. 10.1016/0168-9452(92)90161-E

[51] Gäumann E Naef-Roth S Kern H Zur phytotoxischen Wirksamkeit der Enniatine. J Phytopathol (1960) 40(1):45–51. 10.1111/j.1439-0434.1960.tb01916.x

[52] Burmeister HR Plattner RD Enniatin production by *Fusarium tricinctum* and its effect on germinating wheat seeds. Phytopathology (1987) 77(10):1483–7. 10.1094/Phyto-77-1483

[53] Sy-Cordero AA Pearce CJ Oberlies NH. Revisiting the enniatins: a review of their isolation, biosynthesis, structure determination and biological activities. J Antibiot (Tokyo) (2012) 65(11):541–9. 10.1038/ja.2012.71 22990381 PMC3573854

[54] Benz R Alkali ion transport through lipid bilayer membranes mediated by enniatin A and B and beauvericin. J Membr Biol (1978) 43(4):367–94. 10.1007/BF01871697 581594

[55] Ivanov VT Evstratov AV Sumskaya LV Melnik EI Chumburidze TS Portnova SL Sandwich complexes as a functional form of the enniatin ionophores. FEBS Lett (1973) 36(1):65–71. 10.1016/0014-5793(73)80338-2

[56] Kamyar M Rawnduzi P Studenik CR Kouri K Lemmens-Gruber R. Investigation of the electrophysiological properties of enniatins. Arch Biochem Biophys (2004) 429(2):215–23. 10.1016/j.abb.2004.06.013 15313225

[57] Ovchinnikov YA Ivanov VT Evstratov AV Mikhaleva II Bystrov VF Portnova SL The enniatin ionophores. Conformation and ion binding properties. Int J Pept Protein Res (1974) 6(6):465–98. 10.1111/j.1399-3011.1974.tb02407.x 4455641

[58] Hoornstra D Andersson MA Mikkola R Salkinoja-Salonen MS A new method for in vitro detection of microbially produced mitochondrial toxins. Toxicol In Vitro (2003) 17(5–6):745–51. 10.1016/S0887-2333(03)00097-3 14599472

[59] Trenin AS Tolstykh IV Terekhova LP Zenkova VA Makarova MO Gladkikh EG [The hypolipidemic action of antibiotic 86/88 (enniatin B) in a hepatoblastoma G2 cell culture]. Antibiot Khimioter (2000) 45(4):6–9. 10851642

[60] Hiraga K Yamamoto S Fukuda H Hamanaka N Oda K. Enniatin has a new function as an inhibitor of Pdr5p, one of the ABC transporters in *Saccharomyces cerevisiae*. Biochem Biophys Res Commun (2005) 328(4):1119–25. 10.1016/j.bbrc.2005.01.075 15707993

[61] Dornetshuber R Heffeter P Sulyok M Schumacher R Chiba P Kopp S Interactions between ABC-transport proteins and the secondary *Fusarium* metabolites enniatin and beauvericin. Mol Nutr Food Res (2009) 53(7):904–20. 10.1002/mnfr.200800384 19517454

[62] Devreese M De Baere S De Backer P Croubels S. Quantitative determination of the *Fusarium* mycotoxins beauvericin, enniatin A, A1, B and B1 in pig plasma using high performance liquid chromatography-tandem mass spectrometry. Talanta (2013) 106:212–9. 10.1016/j.talanta.2012.11.068 23598119

[63] Fraeyman S Devreese M Antonissen G De Baere S Rychlik M Croubels S. Comparative oral bioavailability, toxicokinetics, and biotransformation of enniatin B1 and enniatin B in broiler chickens. J Agric Food Chem (2016) 64(38):7259–64. 10.1021/acs.jafc.6b02913 27632250

[64] Meca G Font G Ruiz MJ Comparative cytotoxicity study of enniatins A, A1, A2, B, B1, B4 and J3 on Caco-2 cells, Hep-G2 and HT-29. Food Chem Toxicol (2011) 49:2464–9. 10.1016/j.fct.2011.05.020 21640785

[65] Vejdovszky K Warth B Sulyok M Marko D. Non-synergistic cytotoxic effects of *Fusarium* and *Alternaria* toxin combinations in Caco-2 cells. Toxicol Lett (2016) 241:1–8. 10.1016/j.toxlet.2015.10.024 26529482

[66] Lombardi G Prosperini A Font G Ruiz MJ. Effect of polyphenols on enniatins-induced cytotoxic effects in mammalian cells. Toxicol Mech Methods (2012) 22(9):687–95. 10.3109/15376516.2012.717120 22888764

[67] Uhlig S Gutleb AC Thrane U Flåøyen A. Identification of cytotoxic principles from *Fusarium avenaceum* using bioassay-guided fractionation. Toxicon (2005) 46:150–9. 10.1016/j.toxicon.2005.03.005 15946720

[68] Dornetshuber R Heffeter P Lemmens-Gruber R Elbling L Marko D Micksche M Oxidative stress and DNA interactions are not involved in enniatin- and beauvericin-mediated apoptosis induction. Mol Nutr Food Res (2009) 53(9):1112–22. 10.1002/mnfr.200800571 19653228

[69] Föllmann W Behm C Degen GH. The emerging *Fusarium* toxin enniatin B: in-vitro studies on its genotoxic potential and cytotoxicity in V79 cells in relation to other mycotoxins. Mycotoxin Res (2009) 25(1):11–9. 10.1007/s12550-008-0002-y 23604931

[70] Chou TC Talalay P. Quantitative analysis of dose-effect relationships: the combined effects of multiple drugs or enzyme inhibitors. Adv Enzyme Regul (1984) 22:27–55. 10.1016/0065-2571(84)90007-4 6382953

[71] Ficheux AS Sibiril Y Parent-Massin D. Co-exposure of *Fusarium* mycotoxins: in vitro myelotoxicity assessment on human hematopoietic progenitors. Toxicon (2012) 60(6):1171–9. 10.1016/j.toxicon.2012.08.001 22921581

[72] Juan-García A Manyes L Ruiz MJ Font G. Applications of flow cytometry to toxicological mycotoxin effects in cultured mammalian cells: a review. Food Chem Toxicol (2013) 56:40–59. 10.1016/j.fct.2013.02.005 23422035

[73] Kroemer G Dallaporta B Resche-Rigon M. The mitochondrial death/life regulator in apoptosis and necrosis. Annu Rev Physiol (1998) 60:619–42. 10.1146/annurev.physiol.60.1.619 9558479

